# Gender Differences in the Associations Between Physical Activity, Smartphone Use, and Weight Stigma

**DOI:** 10.3389/fpubh.2022.862829

**Published:** 2022-03-29

**Authors:** Ping Xu, Jung-Sheng Chen, Yen-Ling Chang, Xiaodong Wang, Xingyong Jiang, Mark D. Griffiths, Amir H. Pakpour, Chung-Ying Lin

**Affiliations:** ^1^Department of Educational Psychology, School of Leisure Sports and Management, Guangzhou Sport University, Guangzhou, China; ^2^Department of Medical Research, E-Da Hospital, Kaohsiung, Taiwan; ^3^Department of Family Medicine, Cardinal Tien Hospital, New Taipei, Taiwan; ^4^School of Leisure Sports and Management, Guangzhou Sport University, Guangzhou, China; ^5^Yangan Primary School of Qionglai City, Qionglai, China; ^6^International Gaming Research Unit, Psychology Department, Nottingham Trent University, Nottingham, United Kingdom; ^7^Department of Nursing, School of Health and Welfare, Jönköping University, Jönköping, Sweden; ^8^Institute of Allied Health Sciences, College of Medicine, National Cheng Kung University, Tainan, Taiwan; ^9^Biostatistics Consulting Center, National Cheng Kung University Hospital, College of Medicine, National Cheng Kung University, Tainan, Taiwan; ^10^Department of Public Health, College of Medicine, National Cheng Kung University, Tainan, Taiwan; ^11^Department of Occupational Therapy, College of Medicine, National Cheng Kung University, Tainan, Taiwan

**Keywords:** gender, nomophobia, physical activity, smartphone use, weight stigma

## Abstract

**Background:**

Physical activity (PA) is important for health. However, there is little evidence on how weight stigma, time spent on sedentary activities (including smartphone, social media, online learning), time spent on outdoor activity, and nomophobia associate with PA among Chinese individuals with consideration of gender. The present study examined the aforementioned associations in the COVID-19 pandemic era.

**Methods:**

University students (*N* = 3,135; 1,798 females, 1,337 males) with a mean age of 19.65 years (*SD* = 2.38) years completed an online survey from November to December, 2021. The online survey assessed weight stigma (using the Perceived Weight Stigma Scale and Weight Bias Internalization Scale), PA (using the International Physical Activity Questionnaire Short Form), time spent on different activities (using self-designed items for time on smartphone, outdoor activity, social media, and online learning), and nomophobia (using the Nomophobia Questionnaire). Parallel mediation models were constructed (dependent variable: PA; mediators: perceived weight stigma, weight-related self-stigma, time spent on smartphone, time spent on outdoor activity, time spent on social media, and time spent online learning; independent variable: nomophobia) and evaluated using Hayes' Process Macro Model 4 (IBM SPSS 20.0).

**Results:**

Weight-related self-stigma (β = −0.06; *p* = 0.03), time spent on outdoor activity (β = 0.21; *p* < 0.001), time spent on social media (β = 0.07; *p* = 0.02), time spent on online learning (β = 0.06; *p* = 0.03), and nomophobia (β = −0.07; *p* = 0.01) were all significant factors explaining the PA among female participants. Perceived weight stigma (β = −0.07; *p* = 0.01), time spent on outdoor activity (β = 0.27; *p* < 0.001), and time spent on online learning (β = 0.10; *p* = 0.002) were all significant factors explaining PA among male participants.

**Conclusion:**

Chinese healthcare providers should design programs on weight stigma reduction and outdoor activity improvement to enhance PA among university students.

## Introduction

Physical activity (PA) is important given the robust evidence on its benefits, including (i) cognitive function improvement; (ii) bone health improvement; (iii) weight management; (iv) health risk reduction, such as cancer and diabetes; (v) psychological distress reduction; (vi) sleep quality; (vii) quality of life enhancement; (viii) fall prevention; and (ix) increase in life expectancy (1–4). In contrast, physical inactivity has been found to be associated with increased risk of mental health problems such as depression and physical problems such as cardiovascular diseases (5, 6). Given that university students are at a critical transition moment of human development (e.g., typically moving from high school to university with environment changes), they are likely to decrease their PA as they engage in efforts to cope with the new environment (7, 8). Indeed, a recent survey conducted by the Ministry of Education in mainland China reported that 30% of the university students do not engage in sufficient PA (9). Therefore, it is important for healthcare providers to promote PA among university students and to understand the potential factors contributing to their PA engagement (e.g., weight stigma, time spent on sedentary activities, and time spent on outdoor activities).

Weight stigma has been suggested to be a primary contributor of physical inactivity via lowered motivation to exercise caused by this type of stigma (10–13). Most evidence regarding the effects of weight stigma on physical inactivity is supported by data from Western populations. However, recent studies on Eastern populations (e.g., Chinese) have reported similar effects of weight stigma on physical inactivity (14–16). Moreover, three types of weight stigma have been identified: experienced weight stigma (i.e., individuals experience unfriendly treatment due to their weight), perceived weight stigma (i.e., individuals perceive weight is their problem of causing stigmatized treatment), and weight-related self-stigma (i.e., individuals endorse, agree, and accept that the stigmatized treatments toward them are justified and appropriate) (17). However, effects of different weight stigma types (i.e., perceived weight stigma, experienced weight stigma, and weight-related self-stigma) on physical inactivity have rarely been investigated separately. Therefore, more empirical evidence regarding the different effects of different types of weight stigma on PA (or physical inactivity) is needed.

Apart from weight stigma, time spent on sedentary activities, especially smartphone use and time on social media/learning, may decrease time spent on PA and/or exercise (18–22). It is postulated that when individuals spend more time on sedentary activities (e.g., engaging in social media, using smartphone, or learning), the time spent on physical activities (e.g., exercise) may decrease. However, some evidence indicates that university students may simultaneously be physically active and engaging in substantial sedentary behaviors (23). As a result, mixed findings in the associations between PA and sedentary behaviors have been reported. For example, Fennell et al. (24) found no associations between smartphone use and PA; Towne et al. (25) found positive associations between smartphone use and PA; Grimaldi-and Puyana et al. (26) and Kwok et al. (18) found negative associations between smartphone use and PA using objective measures. Moreover, Lin et al. (19), Shi et al. (20), Van der Velde et al. (21), and Yang et al. (22) all used subjective measures and reported an association between screen time (or problematic smartphone use) and physical inactivity. Therefore, the findings concerning screen use (including smartphone use) and university students' PA are inconsistent and the effects of different type of sedentary screen-based activities may also differ.

Given that university students are grown-ups who are fully in charge of their daily activities and have access to the modern technology, they tend to engage a substantial proportion of daily activities on smartphones (27, 28). In other words, university students depend on smartphone substantially in their daily activities, they are therefore likely to develop nomophobia, a new concept of anxiety resulting from no mobile phone use (i.e., phobia of no mobile phone access) (29, 30). Nomophobia may induce individuals to engage in more smartphone activities (e.g., greater smartphone and social media use) to relieve their anxiety of craving smartphone use (31, 32). Therefore, it is possible that nomophobia serves as a trigger of some sedentary activities and subsequently turns into physical inactivity among university students. Moreover, nomophobia itself may directly decrease PA because evidence shows that anxiety is associated with physical inactivity (33). However, to the best of the present authors' knowledge, no previous study has ever explored the effects of nomophobia on PA or physical inactivity. Therefore, further investigation on this issue is warranted.

Moreover, the COVID-19 pandemic has indirectly increased sedentary behaviors among students given that they have been required to study online instead of studying in a physical classroom (34, 35). In other words, university students have been forced into sedentary lifestyles because of COVID-19. Indeed, most policies to prevent COVID-19 transmission (e.g., lockdowns, quarantining, and public facility closure) decrease individuals' time spent on outdoor activities and increased time on online activities (34, 35). Nevertheless, it is unclear whether individuals have maintained their PA during COVID-19 pandemic. More specifically, individuals' outdoor activity patterns may have changed due to COVID-19 pandemic. Therefore, it is also important to investigate whether the positive relationship between outdoor activity and PA has remained during the COVID-19 pandemic.

Although evidence has shown that PA (or physical inactivity) is associated with weight stigma (10–13), sedentary activity (19–22), and outdoor activity (36), the associations might vary between genders. Prior evidence shows that females as compared with males enjoy PA less and has fewer positive effects on their quality of life (37). Moreover, with the considerations of culture and regulation, especially in East Asia countries (38), females as compared with males are more concerned about their weight in social interaction (39–41). Therefore, it is possible that weight stigma and time spent on social media would be different between genders as evidence showing that girls as compared with boys are more addicted to social media (42). Consequently, the associations between PA and its contributors (i.e., weight stigma, time spent on smartphone, time spent on social media, time spent on online learning, and time spent on outdoor activity) might not always be consistent between genders (43).

**Figure 1:**
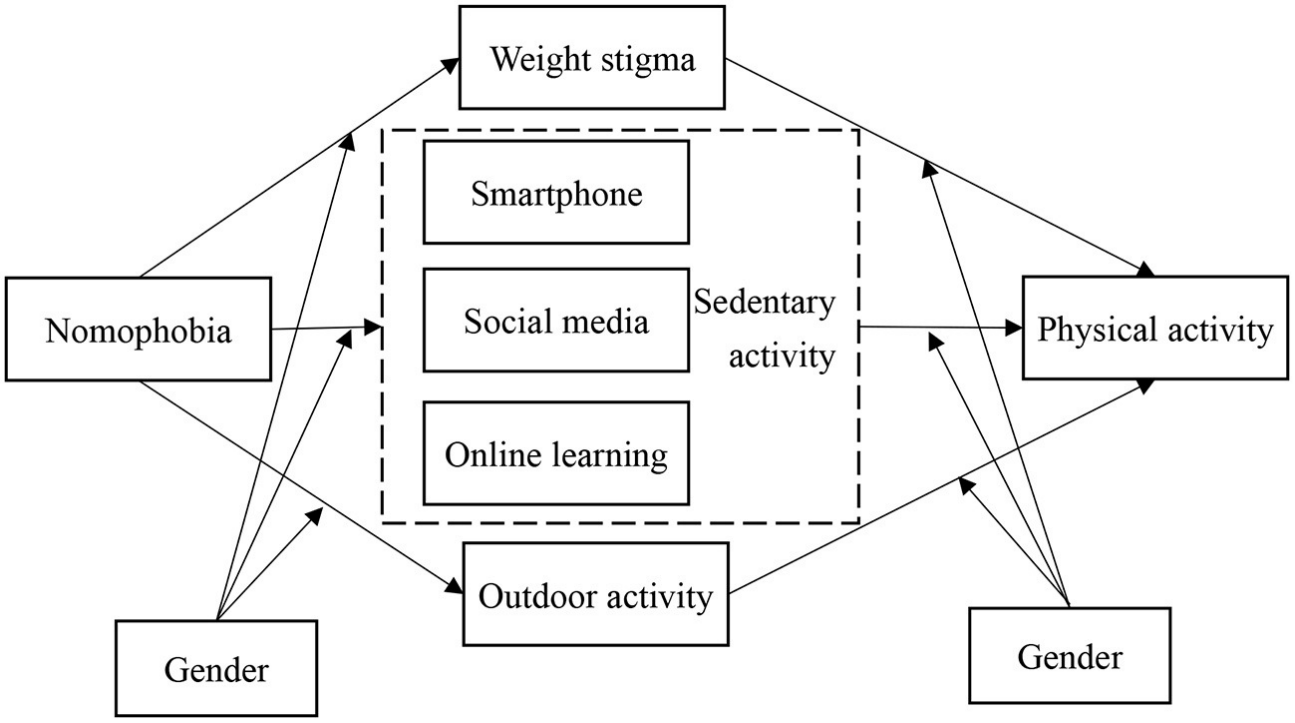
Proposed model in the present study. Nomophobia impacts on physical activity via weight stigma, sedentary activity, and outdoor activity; gender serves as a moderator.

In order to provide contemporary evidence regarding PA engagement among university students, the present study utilized a cross-sectional survey study design to investigate whether weight stigma (including perceived weight stigma and weight-related self-stigma), time on different daily activities (including smartphone use, outdoor activities, social media use, and online learning), and nomophobia were factors contributing to PA (or inactivity during the COVID-19 pandemic). Based on the extant literature, the following hypotheses were proposed: (i) weight stigma would be negatively associated with PA; (ii) time spent on smartphone, social media, and online learning would be negatively associated with PA; (iii) time spent on outdoor activity would be positively associated with PA; (iv) nomophobia would be negatively associated with PA directly and indirectly via weight stigma and time spent on different activities; and (v) the aforementioned associations would be different between male and female participants (Figure 1).

## Methods

### Participants and Data Collection Procedure

The online survey included measures including the Perceived Weight Stigma Scale (PWSS), Weight Bias Internalization Scale (WBIS), International Physical Activity Questionnaire Short Form (IPAQ-SF), and Nomophobia Questionnaire (NMPQ) based on research objectives. Before a formal survey was launched, a pilot study was conducted with 30 university students to assess if the content addressed in the scales were fully understood. In the pilot study, all the students verified that the readability of the survey questions and items were appropriate and understandable. More specifically, the 30 students rated the readability of the survey questions and items and all indicated that the readability was good or very good for all the questions and items used in the survey. Next, an online survey was conducted from November to December 2021 with a total of 26 Chinese universities (Nanchang University, Hangzhou Dianzi University, East China Normal University, Northeast Normal University, Fujian Normal University, Zhengzhou Normal University, Jilin Engineering Normal University, Guangzhou Sport University, Minnan University of Science and Technology, Baoji University of Arts and Sciences, Kaili University, Harbin Cambridge University, Communication University of Shanxi, Guangdong University of Science and Technology, Shanxi Vocational and Technical College, Shenzhen Polytechnic, Shanwei Institute of Technology, Zhejiang Yuying College of Vocational Technology, Ganzhou Teachers College, Guangxi Eco-Engineering Vocational and Technical College, Guangzhou South China Business Trade College, Software Engineering Institute of Guangzhou, Guangzhou College of Commerce, Xiamen Huatian International Vocation Institute, Jiangxi Environmental Engineering Vocational College, and Guizhou Vocational and Technical College of Water Resources and Hydropower). Convenience sampling was utilized which resulted in data being collected from 3,158 participants. All the participants were recruited in the same period (i.e., between November and December 2021). The inclusion criteria for participation were (i) being a university student, (ii) being aged 18 years or above, and (iii) currently residing in mainland China. There were no exclusion criteria. All the data were collected through the online questionnaire *Star* application in China and the *Star* application ensured that each student only completed the survey once. After data cleaning, 3,135 surveys remained for data analysis. The present study was approved by the Human Experimental Ethics Committee in Guangzhou Sport University (Ref no. 2021LCLL-23).

#### Demographics

The participants were asked the following demographics information: (a) age in years; (b) height in cm; (c) weight in kg; (d) marital status reported using single or other; and (e) current disease reported using “yes” or “no”. Self-reported body mass index (BMI) was then calculated using the formula of weight in kilograms divided by squared height in meters.

#### Weight Stigma

Two types weight stigma (i.e., perceived weight stigma and weight-related self-stigma) were assessed using the Perceived Weight Stigma Scale (PWSS) and Weight Bias Internalization Scale (WBIS). The PWSS comprises 10 dichotomous items rated as 1 (*yes*) or 0 (*no*). The 10 item scores are summed (therefore, the maximum score on the PWSS is 10) to represent the level of perceived weight stigma and a higher score indicates a higher level of perceived weight stigma. A sample item for the PWSS is “*People behave as if you are inferior because of your weight status”*. Good psychometric properties of the PWSS have been reported in its factor structure (i.e., unidimensionality is supported) and internal consistency (α = 0.84) (42).

The WBIS contains 11 five-point Likert-scale items rated from 1 (*strongly disagree*) to 5 (*strongly agree*). After reverse coding two item scores (i.e., Items 1 and 9), the 11 item scores are summed (therefore, the maximum score on the WBIS is 55) to represent the level of weight-related self-stigma and a higher score indicates a higher level of weight-related self-stigma (44). A sample item for the WBIS is “*I hate myself because of my weight”*. Good psychometric properties of the WBIS have been reported in its factor structure (i.e., unidimensionality is supported) and internal consistency (α = 0.90) (45, 46).

#### Physical Activity (PA)

PA was assessed using the International Physical Activity Questionnaire Short Form (IPAQ-SF). The IPAQ-SF contains seven items asking how much time in minutes an individual engages in the past week (47). A sample item for the IPAQ-SF is “*During the last 7 days, on how many days did you do vigorous physical activities?”*. Metabolic equivalent of task (MET) was then calculated using the seven IPAQ-SF items and a total MET-minutes in a week. According to the IPAQ-SF guidelines, a specific MET value is given for walking (MET = 3.3), moderate level of PA (MET = 4), and vigorous level of PA (MET = 8). Then, time on each activity is multiplied by the given MET to calculate the MET-minutes per week. For example, if an individual spent (i) one hour swimming (PA at vigorous level) on 3 days, (ii) one hour jogging (PA at moderate level) on 1 day, and (iii) one hour walking on 3 days, the individual would have 2,274 MET-minutes: (8 × 60 min × 3 days) + (4 × 60 min × 1 day) + (3.3 × 60 min × 3 days) = 2,274. Higher MET-minutes in a week indicate higher levels of PA. Psychometric properties of the IPAQ-SF have been supported by the good test-retest reliability (intraclass correlation coefficient = 0.79) (48).

#### Time Spent on Activities

The participants were asked how much time per day they spent on the following four activities in the past week: time spent on smartphone, time spent on outdoor activity, time spent on social media, and time spent on online learning. They were instructed to answer numerically to indicate how many hours and minutes were spent on each activity. A sample item was: “*How much time on average did you spend on your smartphone per day in the last week? Please answer in hours and minutes”*.

#### Nomophobia

Nomophobia was assessed using the Nomophobia Questionnaire (NMPQ). The NMPQ comprises 20 seven-point Likert-scale items rated from 1 (*strongly disagree*) to 7 (*strongly agree*). The 20 item scores are summed (therefore, the maximum score on the NMPQ is 140) to represent the level of nomophobia and a higher score indicates a higher level of nomophobia (49). A sample item for the NMPQ is “*I would feel uncomfortable without constant access to information through my smartphone”*. Good psychometric properties of the NMPQ have been reported in its factor structure (i.e., a four-factor structure is supported) and internal consistency (α = 0.97) (29, 50).

### Data Analysis

First, descriptive statistics (i.e., means and frequencies) were used to understand the participants' characteristics, including their time spent on each activity and the measure scores (e.g., perceived weight stigma and weight-related self-stigma). Independent *t*-tests and χ2 tests were used to examine whether male participants and female participants had significant differences in their characteristics. Two sets of multivariate linear regression models were constructed to examine the potential predictors on PA for different genders separately. In the multivariate linear regression models, the dependent variable was PA (i.e., weekly MET-minutes); the independent variables included perceived weight stigma, weight-related self-stigma, time spent on smartphone, time spent on outdoor activity, time spent on social media, time spent on online learning, and nomophobia. The confounders were age and BMI.

Because it was hypothesized that nomophobia would explain PA via different mediators and such mediation effects might be different between genders, a parallel mediation model (Figure 1) was constructed and tested on male and female participants separately. More specifically, PA was the dependent variable; perceived weight stigma, weight-related self-stigma, time spent on smartphone, on outdoor activity, social media, and online learning were the parallel mediators; nomophobia was the independent variable; and age and BMI were confounders in the parallel mediation models. The mediation effects were examined using the 95% lower limit confidence interval (LLCI) and upper limit confidence interval (ULCI) in the 1,000 bootstrapping resamples conducted using the Hayes' Process Macro Model 4 (51). When the 95% LLCI and ULCI do not cover 0, the mediation effect of that specific mediator is supported (52). All the statistical analyses were performed using the IBM SPSS 20.0 and the level of significance was set at *p* < 0.05 (IBM Corp. Armonk, NY).

## Results

The participants' characteristics are reported in Table 1. The sample (*N* = 3,135) was relatively young (mean age = 19.65 years; *SD* = 2.38) and comprised more female participants (*n* = 1,798; 57.4%). The mean BMI was 23.90 (*SD* = 7.88) for the entire sample. On average, the participants spent 8.27 h daily on their smartphone (*SD* = 4.57), 2.99 h daily on outdoor activities (*SD* = 3.08), 4.04 h daily on social media (*SD* = 3.81), and 3.64 h daily on online learning (*SD* = 3.33). The MET-minutes in a week for the sample was 5,359.25 (*SD* = 4,913.15). Their mean scores were 1.02 for perceived weight stigma (*SD* = 2.22); 26.60 for weight-related self-stigma (*SD* = 8.17); and 75.56 for nomophobia (*SD* = 26.51). In addition, male participants as compared with female participants spent significantly more time on outdoor activity (*p* < 0.001) and had significantly more PA (*p* < 0.001). Female participants as compared with male participants spent significantly more time on smartphone (*p* < 0.001) and social media (*p* < 0.001); and had a higher level of nomophobia (*p* < 0.001). No gender differences were found in the BMI, time spent on online learning, disease, perceived weight stigma, and weight-related self-stigma.

**Table 1:** Participants' characteristics for the entire (*N* = 3,135), female (*n* = 1,798), and male samples (*n* = 1,337).

**Variable**	***n*** **(%) or M (SD)**			***t*** **or χ^2^** **(*p*-value)**
	**Entire sample**	**Female**	**Male**	
**Age** **(year)**	19.65 (2.38)	19.45 (1.95)	19.92 (2.83)	5.14 (<0.001)
**Height** **(cm)**	166.05 (8.33)	161.31 (5.89)	172.44 (6.71)	48.32 (<0.001)
**Weight** **(kg)**	66.08 (22.80)	61.70 (21.38)	71.98 (23.32)	12.79 (<0.001)
**Body mass index** **(kg/m**^**2**^**)**	23.90 (7.88)	23.71 (8.15)	24.15 (7.49)	1.54 (0.12)
**Marital status**				4.14 (0.04)
Single	2,785 (88.8)	1,615 (89.8)	1,170 (87.5)	
Others	350 (11.2)	183 (10.2)	167 (12.5)	
**Time on smartphone** **(hour/day)**	8.27 (4.57)	8.60 (4.46)	7.83 (4.67)	4.53 (<0.001)
**Time on outdoor activity** **(hour/day)**	2.99 (3.08)	2.63 (2.85)	3.47 (3.29)	7.29 (<0.001)
**Time on social media** **(hour/day)**	4.04 (3.81)	4.50 (3.87)	3.42 (3.64)	7.79 (<0.001)
**Time on online learning** **(hour/day)**	3.64 (3.33)	3.60 (3.16)	3.69 (3.55)	0.76 (0.45)
**Physical activity in a week** **(MET-minutes)**	5,359.25 (4,410.28)	4,913.15 (3,850.41)	5,941.25 (4,359.94)	6.45 (<0.001)
**Disease** **(no)**				2.17 (0.14)
No	2,916 (93.0)	1,662 (92.4)	1,254 (93.8)	
Yes	219 (7.0)	136 (7.6)	83 (6.2)	
**Perceived weight stigma**	1.02 (2.22)	0.96 (2.11)	1.09 (2.36)	1.57 (0.12)
**Weight-related self-stigma**	26.60 (8.17)	26.45 (7.76)	26.79 (8.68)	1.13 (0.26)
**Nomophobia**	75.56 (26.52)	80.02 (25.01)	69.55 (27.32)	11.00 (<0.001)

Multivariate linear regression model (R^2^ = 0.12; adjusted R^2^ = 0.11; *F* = 16.42; *p* < 0.001 [male]; R^2^ = 0.09; adjusted R^2^ = 0.09; *F* = 16.18; *p* < 0.001 [female]) indicated that male and female participants had different predictors for their weekly PA (Table 2). More specifically, weight-related self-stigma (standardized coefficient [β] = −0.06; *p* = 0.03), time spent on outdoor activity (β = 0.21; *p* < 0.001), time spent on social media (β = 0.07; *p* = 0.02), time spent on online learning (β = 0.06; *p* = 0.03), and nomophobia (β = −0.07; *p* = 0.01) were significant factors explaining the PA among female participants. Perceived weight stigma (β = −0.07; *p* = 0.01), time on outdoor activity (β = 0.27; *p* < 0.001), and time on online learning (β = 0.10; *p* = 0.002) were significant factors explaining the PA among male participants.

**Table 2:** Multivariate linear regression model in explaining physical activity for female and male participants separately.

	**DV: physical activity in a week (MET-minutes)**	
	**Female**	**Male**
IV	B (SE)/ β (*p*-value)	B (SE)/ β (*p*-value)
Age	**−163.40 (50.60)/ −0.08 (0.001)**	**−176.87 (57.78)/ −0.09 (0.002)**
Body mass index	5.67 (11.86)/ 0.01 (0.63)	−9.52 (16.37)/ −0.02 (0.56)
Perceived weight stigma	−62.31 (45.72)/ −0.04 (0.17)	**−133.86 (53.62)/ −0.07 (0.01)**
Weight-related self-stigma	**−29.44 (13.51)/ −0.06 (0.03)**	−16.92 (17.99)/ −0.03 (0.35)
Time on smartphone	33.55 (25.09)/ 0.04 (0.18)	17.06 (29.59)/ 0.02 (0.56)
Time on outdoor activity	**280.68 (35.89)/ 0.21 (<0.001)**	**363.10 (40.57)/ 0.27 (<0.001)**
Time on social media	**72.14 (30.00)/ 0.07 (0.02)**	−10.44 (40.62)/ −0.01 (0.80)
Time on online learning	**71.31 (32.67)/ 0.06 (0.03)**	**125.25 (40.38)/ 0.10 (0.002)**
Nomophobia	**−10.42 (4.18)/ −0.07 (0.01)**	−5.88 (5.78)/ −0.04 (0.31)

*DV, dependent variable; IV, independent variable*.

*Significant coefficients are in bold*.

Given that nomophobia was not a significant factor explaining PA among male participants, the mediation model was only tested for female participants (Table 3). The mediation model additionally showed that nomophobia had indirect effects on female participants' PA via weight-related self-stigma (unstandardized coefficient [B] = −3.19; 95% CI = −6.2906, −0.4450), time spent on outdoor activity (B = −3.17; 95% CI = −5.4244, −1.4345), and time spent on social media (B = 1.12; 95% CI = 0.0638, 2.4677). Moreover, nomophobia had direct effects on female participants' PA (B = −10.42; 95% CI = −18.6101, −2.2204).

**Table 3:** Mediation model in explaining physical activity for female participants.

			**Coeff. (SE)/ Std. Coeff**.	***p*** **-value**	**LLCI, ULCI**
IV	Mediator	DV			
Nomophobia	Perceived weight stigma	–	**0.01 (0.002)/ 0.10**	**<0.001**	**0.0040, 0.0128**
Nomophobia	Weight-related self-stigma	–	**0.11 (0.008)/ 0.35**	**<0.001**	**0.0937, 0.1232**
Nomophobia	Time on	–	**0.02 (0.005)/ 0.12**	**<0.001**	**0.0120, 0.0302**
Nomophobia	Time on outdoor activity	–	**−0.01 (0.003)/ −0.10**	**<0.001**	**−0.0170, −0.0056**
Nomophobia	Time on social media	–	**0.02 (0.004)/ 0.10**	**<0.001**	**0.0040, 0.0128**
Nomophobia	Time on online learning	–	**−0.01 (0.003)/ −0.11**	**<0.001**	**−0.0195, −0.069**
–	Perceived weight stigma	Physical activity	−62.31 (45.72)/ −0.04	0.17	−151.9896, 27.3609
–	Weight-related self-stigma	Physical activity	**−29.44 (13.50)/ −0.06**	**0.03**	**−55.9312, −2.9499**
–	Time on smartphone	Physical activity	33.55 (25.09)/ 0.04	0.18	−15.6757, 82.7720
–	Time on outdoor activity	Physical activity	**280.68 (35.89)/ 0.21**	**<0.001**	**210.2792, 351.0792**
–	Time on social media	Physical activity	**72.14 (30.00)/ 0.07**	**0.02**	**13.2963, 130.9878**
–	Time on online learning	Physical activity	**71.31 (32.67)/ 0.06**	**0.03**	**7.2218, 135.3995**
Nomophobia	–	Physical activity	**−10.42 (4.18)/ −0.07**	**0.01**	**−18.6101, −2.2204**
Nomophobia	Perceived weight stigma	Physical activity	−0.53 (0.46)/ −0.003	–^a^	−1.3777, 0.4363^a^
Nomophobia	Weight-related self-stigma	Physical activity	**−3.19 (1.50)/ −0.021**	**–** ^a^	**−6.2906, −0.4450** ^a^
Nomophobia	Time on smartphone	Physical activity	0.71 (0.65)/ 0.005	–^a^	−0.3737, 2.1419^a^
Nomophobia	Time on outdoor activity	Physical activity	**−3.17 (1.06)/ −0.021**	**–** ^a^	**−5.4244, −1.4345** ^a^
Nomophobia	Time on social media	Physical activity	**1.12 (0.62)/ 0.007**	**–** ^a^	**0.0638, 2.4677** ^a^
Nomophobia	Time on online learning	Physical activity	−0.94 (0.58)/ −0.006	–^a^	−2.1970, 0.0982^a^

*Age and body mass index were controlled in the model*.

a *Calculated using bootstrapping method (1,000 bootstrapping resamples); therefore, no p-values reported*.

*IV, independent variable; DV, dependent variable; Coeff., coefficient; Std., standardized; SE, standard error; LLCI, lower limit confidence interval at 95%; ULCI, upper limit confidence interval at 95%*.

*Significant coefficients are in bold*.

## Discussion

The first hypothesis in the present study was partially supported given that weight stigma was negatively associated with PA engagement in different ways. More specifically, PA was negatively associated with perceived weight stigma but not weight-related self-stigma among male participants; PA was negatively associated with weight-related self-stigma but not perceived weight stigma among female participants. The second hypothesis was not supported given that time spent on smartphone was not associated with PA; time spent on social media was positively associated with PA among female participants; and time spent on online learning was positively associated with PA among both genders. The third hypothesis was fully supported because outdoor activity was positively associated with PA. The fourth hypothesis was partially supported because nomophobia was negatively and directly associated with PA among females but not among males. Moreover, nomophobia was negatively associated with PA via weight-related self-stigma and time spent on outdoor activity but not other proposed mediators among females. The fifth hypothesis was fully supported because different associations were found between males and females.

Consistent with prior literature findings (10–13), weight stigma in the present study was found to be negatively associated with PA. Previous research suggests that weight stigma is a variable that lowers individuals' motivations to exercise and subsequently results in low levels of PA among individuals (10–13). Indeed, Cheng et al. (14) and Fung et al. (15) found that Hong Kong university students were likely to have weight stigma effects in relation to their intention to engage in PA. Because individuals having weight stigma (either weight-related self-stigma or perceived weight stigma) may want to escape from other individuals' judgements, one coping strategy that is employed is not to exercise in front of them (10–15). More specifically, those who have weight stigma issues may feel like they are being laughed at and/or derided by others when they exercise. Therefore, they may consider that PA engagement may increase their risk of being laughed at and/or derided. As a result, their weight stigma may result in increased physical inactivity.

The present findings extend the knowledge regarding the association between weight stigma and PA in relation to gender differences. Female university students were impacted more by their weight-related self-stigma on their PA, while male students were more impacted by the perceived weight stigma on PA. The gender differences may be explained by the features of gender such as females have more internalizing problems (e.g., depression) and males having more externalizing problems (e.g., aggressive behaviors) (53–59). Internalizing problems among females may maximize their feelings of weight-related self-stigma, which in turn, are associated with their lowered PA (59). Externalizing problems among males may make them share the attitudes and behaviors of their peers, which strengthens the effects of their perceived weight stigma on PA (53–58).

Contrary to the hypothesis, the present study found that time spent on smartphone did not associate with PA. Inconsistent findings have previously been found for the associations between time spent on smartphone and PA (24–26). Several reasons may explain the inconsistent findings. First, objective measures and subjective measures assessing time spent on smartphone and PA may result in different findings. Indeed, the present study used self-reports and have similar findings to another study using self-reports (24). Second, Peterson et al. (23) noted that university students may use smartphone apps to record and monitor their PA. Moreover, they may also use smartphone apps to help them facilitate PA (e.g., use of a “reminder” function to help them to engage in PA, calculating energy expenditure in exercising). Therefore, some smartphone use may be directly associated with PA. Therefore, it is possible that some participants in the present study used smartphones and were physically active, which resulted in the nonsignificant association between time spent on smartphone and PA. Third, some university students may use smartphone apps to engage in exercise given many exercise apps have been developed (60, 61). Therefore, the association between time spent on smartphone and PA could be varied depending on how university students used their smartphone.

Contrary to two other hypotheses, time spent on online learning (for both genders) and time spent on social media (for females) were positively associated with PA. A possible explanation for the positive association between time spent on online learning and PA is that the students who spent time on online learning may be more diligent, which could be an important factor in pushing them to do exercise. However, the present study does not have evidence to support this speculation and future studies are needed to verify this. Regarding the positive association between time spent on social media and PA among female participants, the explanation may be that female participants use social media to relieve their emotional distress (62), which in turn puts them in a better mood to exercise. However, this speculation also needs further study to corroborate.

The positive associations between outdoor activity and PA among both genders supported the hypothesis. These findings echo previous PA advocators that it is important to push university students to go outdoors instead of staying indoors (36). Given that the present findings were obtained during COVID-19 pandemic, the implication is that government and policymakers may consider implemented initiatives allowing citizens to go outdoors if lockdown and closures are needed for controlling COVID-19 transmission. However, such initiatives would need to be carefully discussed so that public health safety is maintained.

Negative effects of nomophobia on PA were found among females but not among males, which partially supported the hypothesis. The effects among females but not males may again be explained by the fact that females as compared with males are more likely to (i) have internalizing problems (e.g., depression) (60, 61), and (ii) use more social media to express emotions (62). Female participants in the present study may have relied more on smartphones than male participants as evidenced by their higher NMPQ score (Table 1). As a result, nomophobia may have effects on PA among females but not males. Additionally, mediated roles of weight-related self-stigma, time spent on outdoor activity, and time spent on social media in the association between nomophobia and PA engagement were only found among females.

There are some limitations in the present study. First, the present study only used subjective measures to assess all the factors. Prior evidence shows that there could be some differences between objective measures and subjective measures in PA and time spent on smartphone (26, 34). Moreover, social desirability and recall bias may make participants report better performance in PA and smartphone use. Therefore, the present findings are subject to the biases caused by self-reports (42). Second, the present study was conducted using online survey with convenience sampling. Therefore, the present findings were not from a representative sample. Third, the cross-sectional study design used in the present study provides weak evidence in causality between nomophobia, weight stigma, time spent on different activities, and PA engagement. Therefore, the directions and mediations proposed and examined in the present study need future studies to corroborate using a longitudinal study design.

## Conclusion

The present study found that factors regarding PA engagement were different among male and female mainland Chinese university students. More specifically, younger age, more time spent on outdoor activity, and more time spent on online learning were significantly associated with higher levels of PA for both genders. Among female participants, PA engagement was negatively associated with weight-related self-stigma and nomophobia but positively associated with time spent on social media. The positive association between PA engagement and time spent on social media suggests that social media platforms could be motivational tools in promoting a physically active lifestyle among young female adults. Moreover, the effect between nomophobia and PA engagement was indirect via weight-related self-stigma, time spent on outdoor activity, and time spent on social media. Among male participants, PA engagement was negatively associated with perceived weight stigma. Therefore, Chinese healthcare providers should design programs for weight stigma reduction and outdoor activity improvement to enhance PA among university students.

## Data Availability Statement

The raw data supporting the conclusions of this article will be made available by the authors, without undue reservation.

## Ethics Statement

The studies involving human participants were reviewed and approved by the Human Experimental Ethics Committee in Guangzhou Sport University (Ref no. 2021LCLL-23). Written informed consent for participation was not required for this study in accordance with the national legislation and the institutional requirements.

## Author Contributions

PX, J-SC, XW, and Y-LC contributed to the study conception and design. PX, Y-LC, and C-YL performed material preparation. PX, XW, and XJ collected data. Y-LC analyzed data. PX, J-SC, MG, AP, and C-YL interpreted data. PX and Y-LC wrote the first draft of the manuscript. J-SC, XW, XJ, Y-LC, MG, AP, and C-YL critically reviewed the manuscript. MG was responsible for all final editing. All authors have read and approved the final version of the manuscript, and agree with the order of presentation of the authors. All authors contributed to the article and approved the submitted version.

## Funding

This work was supported by the Ministry of Science and Technology, Taiwan (MOST 110-2410-H-006-115), the Higher Education Sprout Project, Ministry of Education to the Headquarters of University Advancement at National Cheng Kung University (NCKU), the 2021 Southeast and South Asia and Taiwan Universities Joint Research Scheme (NCKU 31), 2021 Guangdong Province Educational Science Planning Special Project for Higher Education Research on Improving the Teaching Ability of Physical Education Teachers in Colleges and Universities under the Background of Sports and Medicine Integration-Taking Guangdong-Hong Kong-Macao Greater Bay Area Colleges and Universities as an Example (2021GXJK619), and the E-Da hospital (EDAHT111032).

## Conflict of Interest

CY-L was employed by Biostatistics Consulting Center. The remaining authors declare that the research was conducted in the absence of any commercial or financial relationships that could be construed as a potential conflict of interest.

## Publisher's Note

All claims expressed in this article are solely those of the authors and do not necessarily represent those of their affiliated organizations, or those of the publisher, the editors and the reviewers. Any product that may be evaluated in this article, or claim that may be made by its manufacturer, is not guaranteed or endorsed by the publisher.
